# Hormonal sensitivity of mood symptoms in women with ADHD across the lifespan

**DOI:** 10.1192/j.eurpsy.2023.92

**Published:** 2023-07-19

**Authors:** J. S. Kooij

**Affiliations:** ^1^Psychiatry, AUMC/VUMc, Amsterdam; ^2^Adult ADHD, PsyQ, the Hague, Netherlands

## Abstract

**Abstract:**

EPA 2023SP-1290

Objective

In medicine, women are still understudied because they are considered less reliable research subjects than men, due to hormonal changes during the lifespan. Women with ADHD have been even more understudied, while exactly their hormonal mood changes and increased severity of ADHD urgently need our research attention.

Methods

In a selfreport questionnaire study (Dorani 2021) among 209 women with ADHD, hormonal mood changes during the menstrual cycle, after childbirth and during menopausal transition were investigated, and compared with available data from women of the general population, using the same instruments.

Results

The data showed that in every episode of hormonal changes, women with ADHD suffered from a 2-3 fold increase in frequency and severity of mood changes.

Conclusions

This first study points to increased severity of mood changes and probably also ADHD symptoms during episodes of hormonal changes in women with ADHD during the lifespan. During this talk, the findings and their etiological background will be clarified, such as interaction between the sex hormones estrogen and progesteron with dopamine and other neurotransmitters in the brain. Treatment options will be discussed as well.

**Disclosure of Interest:**

None Declared

